# Japanese subgroup analysis of EV‐301: An open‐label, randomized phase 3 study to evaluate enfortumab vedotin versus chemotherapy in subjects with previously treated locally advanced or metastatic urothelial carcinoma

**DOI:** 10.1002/cam4.5165

**Published:** 2022-09-02

**Authors:** Nobuaki Matsubara, Junji Yonese, Takahiro Kojima, Haruhito Azuma, Hiroaki Matsumoto, Thomas Powles, Jonathan E. Rosenberg, Daniel P. Petrylak, Maria Matsangou, Chunzhang Wu, Mary Campbell, Mayumi Yamashiro

**Affiliations:** ^1^ Department of Medical Oncology National Cancer Center Hospital East Chiba Japan; ^2^ Department of Urology Cancer Institute Hospital, Japanese Foundation for Cancer Research Tokyo Japan; ^3^ Department of Urology Aichi Cancer Center Nagoya Aichi Japan; ^4^ Department of Urology Osaka Medical and Pharmaceutical University Osaka Japan; ^5^ Department of Urology Yamaguchi University, School of Medicine Ube Japan; ^6^ Barts Cancer Institute, CRUK Experimental Cancer Medicine Centre London UK; ^7^ Department of Medicine, Division of Solid Tumor Oncology Genitourinary Oncology Service, Memorial Sloan Kettering Cancer Center New York New York USA; ^8^ Yale Cancer Center New Haven Connecticut USA; ^9^ Astellas Pharma, Inc. Northbrook Illinois USA; ^10^ Seagen Inc. Bothell Washington USA; ^11^ Astellas Pharma, Inc. Tokyo Japan

**Keywords:** antibody‐drug conjugate, enfortumab vedotin, Japanese, metastatic, urothelial carcinoma

## Abstract

**Background:**

Enfortumab vedotin (EV) is an antibody‐drug conjugate showing significant overall survival (OS) benefit versus chemotherapy for patients with previously treated locally advanced or metastatic urothelial carcinoma (la/mUC) in EV‐301. This subgroup analysis was conducted to further analyze the efficacy and safety in a Japanese population.

**Methods:**

In the open‐label, phase 3 EV‐301 trial, patients with la/mUC were randomized 1:1 to EV 1.25 mg/kg on Days 1, 8, and 15 for 28‐day cycles or investigator‐preselected standard chemotherapy (SC; docetaxel or paclitaxel for patients in Japan) on Day 1 of each 21‐day cycle. Primary endpoint was OS and secondary efficacy endpoints included progression‐free survival (PFS) and overall response rate (ORR). Safety/tolerability was also evaluated.

**Results:**

As of the July 15, 2020 cut‐off date for the interim analysis, the Japanese subgroup included 86 patients (EV: *n* = 36; SC: *n* = 50). Median OS was 15.18 months for EV and 10.55 months for SC (HR: 0.437 [95% CI: 0.209, 0.914]). Median PFS was 6.47 months for EV and 5.39 months for SC (HR: 0.464 [95% CI: 0.258, 0.835]). Confirmed ORR was 34.4% for EV and 21.3% for SC. A higher proportion of patients receiving SC versus EV had treatment‐related adverse events (TRAEs; 97.9% vs. 91.7%, respectively), including grade ≥ 3 TRAEs (75.0% vs. 63.9%).

**Conclusions:**

This subgroup analysis confirmed that EV, with consistent efficacy and safety/tolerability in the EV‐301 Japanese subgroup and overall study population, represents an important treatment option for previously treated patients with la/mUC.

## Graphical abstract

In the global, open‐label, phase 3 EV‐301 trial, enfortumab vedotin (EV) demonstrated clinically significant overall survival (OS) benefit compared with standard chemotherapy in patients with previously treated locally advanced or metastatic urothelial carcinoma. This subgroup analysis of EV‐301 Japanese patients confirmed that EV demonstrated an OS benefit and no new safety signals identified in the Japanese subpopulation, consistent with the efficacy and safety/tolerability in the EV‐301 overall study population.

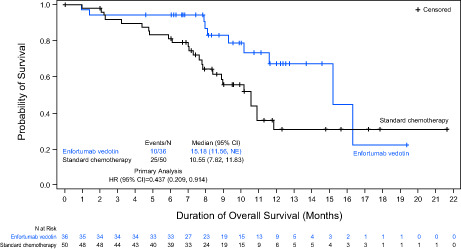

## 1 INTRODUCTION

Globally, bladder cancer ranks 10th as one of the most commonly diagnosed cancers, and an estimated 213,000 deaths occurred in 2020.^1^ Bladder cancer is the 13th leading cause of cancer in Japan.^2^ Of 378,500 projected cancer deaths in 2021 in Japan, an estimated 10,000 will be due to bladder cancer.^3^ The most common histological type of bladder cancer is urothelial carcinoma (UC).^4^ The reported median survival for patients worldwide and in Japan with recurrent or metastatic UC treated with platinum‐containing chemotherapy and/or PD‐1/L1 inhibitors remains under 18 months, and there is a clear, unmet medical need in this setting.^5^, ^6^, ^7^, ^8^

Enfortumab vedotin (EV) is an antibody‐drug conjugate comprised of a fully human monoclonal antibody directed against Nectin‐4 and monomethyl auristatin E (MMAE), a microtubule‐disrupting agent, attached via a protease‐cleavable linker; MMAE release leads to cell cycle arrest and death.^9^, ^10^ In the confirmatory, randomized, phase 3 EV‐301 trial (NCT03474107), EV showed clinically significant overall survival (OS) benefit compared with investigator‐chosen standard chemotherapy (SC; docetaxel, paclitaxel, or vinflunine) in patients with previously treated locally advanced or metastatic urothelial carcinoma (la/mUC) (HR: 0.70 [95% CI: 0.56, 0.89], *p* = 0.001).^11^ Subsequently, in July 2021, EV received regular approval from the United States Food and Drug Administration for the treatment of adults with la/mUC who previously received a PD‐1/L1 inhibitor and a platinum‐based chemotherapy and has been approved by Japan's Ministry of Health, Labour, and Welfare in September 2021 based upon the findings of the global EV‐301 trial.^10^, ^12^, ^13^, ^14^

While avelumab is approved for maintenance treatment of patients with la/mUC and pembrolizumab is approved for patients with UC who have progressed during or following platinum‐containing chemotherapy, there are currently no guideline‐recommended treatment options in Japan that have demonstrated a survival benefit for patients with previously treated advanced UC, including after failure of PD‐1/L1 inhibitor therapy.^15^, ^16^, ^17^, ^18^, ^19^ Compared to the United States and countries in the European Union, fewer approved options are available regardless of treatment line and eligibility for cisplatin in Japan. As such, effective treatments for patients who have progressed beyond platinum‐based chemotherapy and PD‐1/L1 inhibitor treatment are an unmet need. Because certain anticancer agents have shown differences in the safety profile or tolerability between Asian and Caucasian patients, the comparison of safety data between these populations might be informative for clinicians in daily practice.^20^, ^21^ We present a subgroup analysis of patients in Japan in the EV‐301 trial to further analyze efficacy and safety in a Japanese population.

## 2 METHODS

Patients at approximately 185 study centers in North America, Europe, Asia Pacific, and Latin America underwent 1:1 randomization to receive EV or SC. Randomization was performed using interactive response technology, with stratification for the following variables: Eastern Cooperative Oncology Group (ECOG) performance status, regions of the world, and liver metastasis. EV 1.25 mg/kg via intravenous (IV) infusion over 30 min was given on Days 1, 8, and 15 for continuous 28‐day cycles. Standard chemotherapy was preselected by the investigator prior to randomization, including the following options: docetaxel 75 mg/m^2^ IV over 60 min, vinflunine 320 mg/m^2^ IV over 20 min (where approved for UC and capped at 35%), and paclitaxel 175 mg/m^2^ via IV infusion over 3 h on Day 1 of each 21‐day cycle. For patients in Japan, either docetaxel or paclitaxel was chosen since vinflunine has not been approved in Japan. Dose modifications and interruptions were permitted to manage adverse events (AEs) based on prespecified criteria.

Key eligibility criteria included adult patients with histologically or cytologically confirmed UC (including those with squamous cell differentiation or mixed cell types) and radiologically documented metastatic or locally advanced disease. Patients who experienced radiographic progression or relapse during or after a PD‐1/L1 inhibitor for locally advanced or metastatic disease, patients who had received a platinum‐containing regimen for la/mUC, for use in the neoadjuvant or adjuvant setting (progression within 12 months of completion if administered in the adjuvant or neoadjuvant setting), and patients who had an ECOG status of 0 or 1 were eligible; patients who discontinued PD‐1/L1 inhibitor treatment due to toxicity were eligible for the study if they had evidence of disease progression after discontinuation. Patients with a preexisting grade ≥ 2 sensory or motor neuropathy or ongoing clinically significant toxicity (grade 2 or higher) associated with prior treatment, active central nervous system metastases, uncontrolled diabetes, and active keratitis or corneal ulcerations were excluded. Patients received treatment until radiographic disease progression was determined per investigator assessment, other discontinuation criterion had been met, or until study completion or termination, whichever occurred first. Additional details of the EV‐301 study have previously been published.^11^

The primary endpoint was OS, defined as the time from randomization to the date of death. Secondary efficacy endpoints included progression‐free survival (PFS; radiological disease progression or death from any cause, whichever occurred first, from the time of randomization), overall response rate (ORR; proportion of patients with complete and partial response), duration of response (DoR; time from the date of first response), and disease control rate (DCR; proportion of patients with complete and partial response and stable disease). Imaging was performed at baseline and every 56 (±7) days from the first dose of the study treatment throughout the study until PFS was documented by radiological disease progression, the patient was lost to follow‐up, died, withdrew study consent, or started subsequent anticancer treatment, whichever occurred earlier. Response Evaluation Criteria in Solid Tumors (RECIST) version 1.1 was used to assess PFS, ORR, DoR, and DCR. All patients had scintigraphy performed at screening/baseline and those with a positive scan received scintigraphy at least every 56 (±7) days. Brain scans were performed, if clinically indicated, at baseline and throughout the study. Safety was also evaluated as a secondary endpoint.

OS and PFS were analyzed using the intention‐to‐treat (ITT) population, consisting of all randomized patients. Cox proportional hazards model was used to estimate risk of death and/or relapse, Kaplan–Meier methods were used to estimate median survival by treatment arm, and a log‐rank test was used to compare the primary efficacy endpoint of OS.^11^ Prespecified interim analyses were conducted using the O'Brien‐Fleming boundaries per Lan‐DeMets methods, as previously described.

ORR and DCR were analyzed in patients from the ITT population with measurable disease at baseline and ≥ 6 months of follow‐up (i.e., response‐evaluable set) using a Cochran–Mantel–Haenszel test to estimate differences in response rates between treatment arms. DoR was also analyzed using the Kaplan–Meier method. Safety analyses were performed in all patients receiving any study drug (i.e., safety analysis set) and evaluated using descriptive statistics to summarize the frequency of AEs. This analysis in the Japanese subpopulation was conducted post hoc and all analyses were unstratified. All data processing, summarization, and analyses were performed using SAS^®^ Version 9.2 or higher.

## 3 RESULTS

### 3.1 Patient demographics, baseline clinical characteristics, and disposition

**FIGURE 1:**
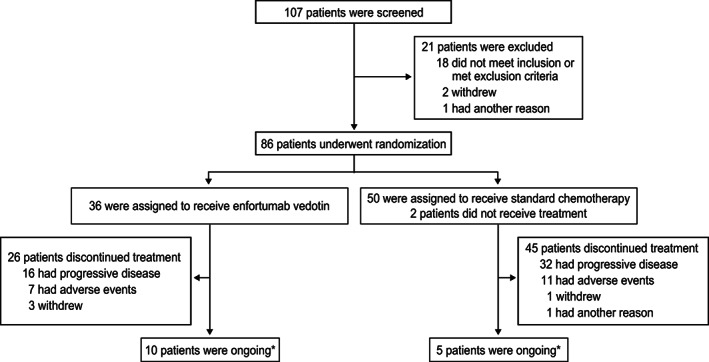
Patient disposition. *The number of patients who were still on treatment as of the data cut‐off date of July 15, 2020.

The data cut‐off for the interim analysis was July 15, 2020. The Japanese subgroup included 86 patients randomized to EV (*n* = 36) and SC (*n* = 50) from 25 sites in Japan (Figure 1). All patients assigned to EV received study treatment. Most patients assigned to SC received study treatment except for two patients (treatment not received for the reasons: due to patient withdrawal [*n* = 1] and “other” reason [*n* = 1]); in the SC group, 36 patients received docetaxel and 12 patients received paclitaxel. The majority of patients in the overall Japanese subgroup were male (74.4%) and the median age was 68.5 years (range: 44 to 87 years); median age was 70.0 years in the EV group and 66.5 in the SC group (Table 1). The proportion of patients with liver metastasis was 13.9% in the EV group and 22.0% in the SC group; visceral metastasis was present in 83.3% and 81.6% of patients, respectively. The most common reasons for treatment discontinuation in both treatment groups were progressive disease (EV: 44.4% and SC: 64.0%) and AEs (EV: 19.4% and SC: 22.0%). Overall, baseline patient characteristics were similar between the Japanese subpopulation and overall EV‐301 population.

**TABLE 1:** Patient demographics and baseline clinical characteristics of the Japanese subgroup (intention‐to‐treat population)

Parameter/characteristic	Enfortumab vedotin (*n* = 36)	Standard chemotherapy (*n* = 50)
Age, years		
Mean (SD)	69.86 (5.93)	67.06 (9.07)
Median (range)	70.00 (58.0, 81.0)	66.50 (44.0, 87.0)
Age category (years), *n* (%)		
<65	7 (19.4)	22 (44.0)
65 to <75	22 (61.1)	17 (34.0)
≥75	7 (19.4)	11 (22.0)
Sex, *n* (%)		
Male	28 (77.8)	36 (72.0)
Female	8 (22.2)	14 (28.0)
Tobacco use, *n* (%)		
Former user	24 (66.7)	30 (60.0)
Current user	2 (5.6)	3 (6.0)
Never used	10 (27.8)	16 (32.0)
Not reported	0	1 (2.0)
History of diabetes/hyperglycemia, *n* (%)	5 (13.9)	9 (18.0)
ECOG performance status, *n* (%)		
0	30 (83.3)	31 (62.0)
1	6 (16.7)	19 (38.0)
Bellmunt risk score, *n* (%)		
0–1	33 (91.7)	36 (72.0)
≥2	3 (8.3)	14 (28.0)
Primary tumor site, *n* (%)		
Upper tract (renal pelvis, ureter)	22 (61.1)	25 (50.0)
Bladder/other	14 (38.9)	25 (50.0)
Sites of metastasis, *n* (%)		
Lymph node only	3 (8.3)	5 (10.2)
Visceral site	30 (83.3)	40 (81.6)
Liver metastasis	5 (13.9)	11 (22.0)
Histology type at diagnosis, *n* (%)		
UC/transitional cell	36 (100)	44 (88.0)
UC mixed	0	5 (10.0)
Other	0	1 (2.0)
Previous systemic therapies, *n* (%)		
1–2	28 (77.8)	44 (88.0)
≥3	8 (22.2)	6 (12.0)
Type of prior platinum‐based treatment received, *n* (%)		
Cisplatin‐based only	22 (61.1)	32 (64.0)
Carboplatin‐based only	7 (19.4)	10 (20.0)
Both cisplatin‐based and carboplatin‐based	7 (19.4)	7 (14.0)
Median time since diagnosis of metastatic or locally advanced disease (range), mo	18.3 (0.4, 46.0)	13.3 (0.7, 53.8)

Abbreviations: ECOG, Eastern Cooperative Oncology Group; SD, standard deviation; UC, urothelial carcinoma.

### 3.2 Efficacy

**FIGURE 2:**
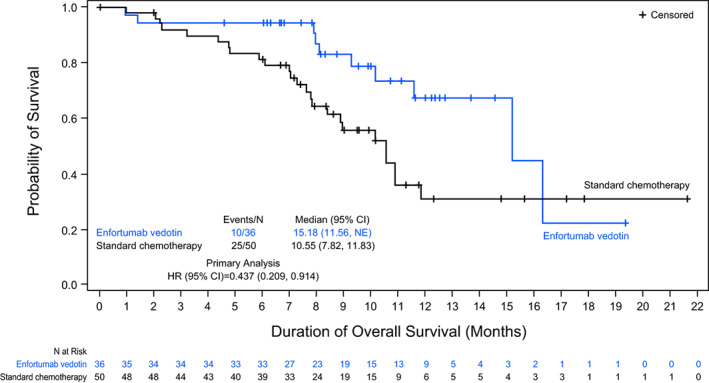
Overall survival in the Japanese subgroup (intention‐to‐treat population).

At data cut‐off, 35 deaths had been observed (10 in the EV group and 25 in the SC group) and the median follow‐up time was 10.71 months in the Japanese subgroup. The median duration of treatment at data cut‐off was 5.59 months (range: 0.9–19.4) in the EV group and 4.01 months (range: 0.7–11.7) in the SC group. In an unstratified analysis, the risk of death was 56% lower with EV than with SC (HR: 0.437 [95% CI: 0.209, 0.914]; Figure 2). The median OS was 15.18 months in the EV group and 10.55 months in the SC group. At 12 months, the OS rate was 67.4% in the EV group and 30.9% in the SC group.

**FIGURE 3:**
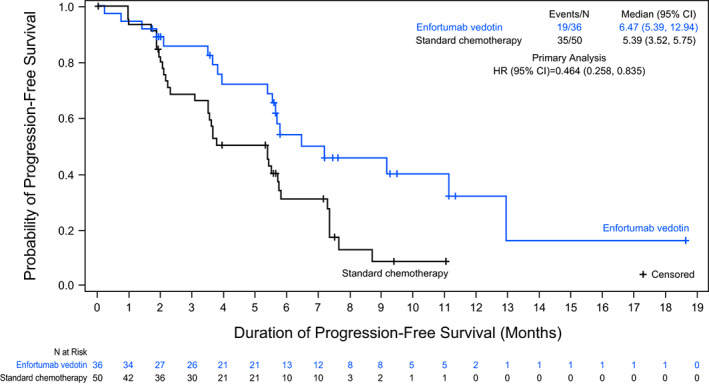
Progression‐free survival in the Japanese subgroup (intention‐to‐treat population).

PFS was also prolonged in the EV group compared with the SC group (HR: 0.464 [95% CI: 0.258, 0.835]; Figure 3). The median PFS was 6.47 months and 5.39 months in the EV and SC groups, respectively.

Confirmed ORR was higher in the EV group than in the SC group (34.4% [95% CI: 18.57, 53.19] vs. 21.3% [95% CI: 10.70, 35.66]) (Table 2). Complete response was observed in 6.3% (*n* = 2) of patients in the EV group and in no patients in the SC group; additional response data is shown in Table 2. Confirmed DCR was higher in the EV (84.4% [95% CI: 67.21, 94.72]) vs. SC group (63.8% [95% CI: 48.52, 77.33]). The median DoR per investigator assessment in patients with confirmed complete and partial response was 5.59 months in both the EV (*n* = 11) and SC (*n* = 10) groups.

**TABLE 2:** Summary of confirmed response^a^ in the Japanese subgroup (response‐evaluable population)

Category	Enfortumab vedotin (*n* = 32)	Standard chemotherapy (*n* = 47)
Best overall response, confirmed^b^, *n* (%)		
CR	2 (6.3)	0
PR	9 (28.1)	10 (21.3)
Stable disease	16 (50.0)	20 (42.6)
Progressive disease	4 (12.5)	10 (21.3)
Not evaluable	1 (3.1)	7 (14.9)
ORR, confirmed (CR or PR), *n* (%)	11 (34.4)	10 (21.3)
95% CI for ORR, (%)	(18.57, 53.19)	(10.70, 35.66)
DCR, confirmed^c^, *n* (%)	27 (84.4)	30 (63.8)
95% CI for DCR^c^, (%)	(67.21, 94.72)	(48.52, 77.33)
DoR, months		
Median (95% CI), months	5.59 (3.71, 9.46)	5.59 (3.48, NE)
Range	3.52, 9.46	3.48, 8.97^d^

Abbreviations: CI, confidence interval; CR, complete response; DCR, disease control rate; DoR, duration of response; NE, not estimable; ORR, overall response rate; PR, partial response; RECIST, Response Evaluation Criteria in Solid Tumors.

^a^ The definition of best overall response followed RECIST v1.1.

^b^ CR/PR must have been confirmed by two scans a minimum of 4 weeks apart. The minimum duration for stable disease was 7 weeks.

^c^ DCR was defined as the proportion of patients who had a best overall response of confirmed CR, confirmed PR, or stable disease (≥7 weeks).

^d^ Indicates censoring.

### 3.3 Safety/tolerability

The incidence of treatment‐related AEs (TRAEs) was numerically higher in the SC group (97.9%) compared with the EV (91.7%) group (Table 3). Clinically significant adverse reactions noted in Japan's prescribing information for EV and reported in Japanese patients in this analysis included severe skin disorders, hyperglycemia, peripheral neuropathy, myelosuppression, infection, and renal impairment.^22^ Grade ≥ 3 TRAEs occurred in 63.9% of patients in the EV group and 75.0% of patients in the SC group. After adjustment for treatment exposure, rates of grade ≥ 3 TRAEs were 3.5 events per patient‐year in the EV group and 11.9 events per patient‐year in the SC group. Grade ≥ 3 TRAEs occurring in at least 10% of patients in the Japanese subgroup included decreased neutrophil count (13.9%) and drug eruption (11.1%) in the EV group and decreased neutrophil count (45.8%), white blood cell count (27.1%), febrile neutropenia (12.5%), and decreased lymphocyte count (10.4%) in the SC group. TRAEs leading to dose reduction, interruption of treatment, or withdrawal of treatment occurred in 38.9%, 63.9%, and 25.0% of patients in the EV group, respectively, and in 33.3%, 29.2%, and 22.9% of patients in the SC group, respectively. AEs resulting in death, excluding disease progression, during the treatment period and regardless of relatedness to study treatment, occurred in two patients in the EV group (abnormal hepatic function, *n* = 1 and dyspnea, *n* = 1) and no patients in the SC group.

**TABLE 3:** Treatment‐related adverse events (safety population)^a^

Adverse event, *n* (%)	Enfortumab vedotin (*n* = 36)		Standard chemotherapy (*n* = 48)	
	Any grade	Grade ≥ 3	Any grade	Grade ≥ 3
Overall	33 (91.7)	23 (63.9)	47 (97.9)	36 (75.0)
Alopecia	19 (52.8)	0	18 (37.5)	0
Dysgeusia	18 (50.0)	0	6 (12.5)	0
Peripheral sensory neuropathy	17 (47.2)	0	15 (31.3)	0
White blood cell count decreased	9 (25.0)	2 (5.6)	18 (37.5)	13 (27.1)
Decreased appetite	9 (25.0)	2 (5.6)	10 (20.8)	1 (2.1)
Pruritus	9 (25.0)	1 (2.8)	3 (6.3)	0
Neutrophil count decreased	8 (22.2)	5 (13.9)	24 (50.0)	22 (45.8)
Anemia	8 (22.2)	2 (5.6)	9 (18.8)	4 (8.3)
Rash maculo‐papular	8 (22.2)	1 (2.8)	2 (4.2)	0
Drug eruption	6 (16.7)	4 (11.1)	1 (2.1)	1 (2.1)
Lymphocyte count decreased	6 (16.7)	2 (5.6)	5 (10.4)	5 (10.4)
Amylase increased	4 (11.1)	2 (5.6)	0	0
Malaise	4 (11.1)	0	15 (31.3)	0
Pyrexia	3 (8.3)	2 (5.6)	10 (20.8)	0
Lipase increased	3 (8.3)	2 (5.6)	1 (2.1)	1 (2.1)
Rash erythematous	2 (5.6)	2 (5.6)	1 (2.1)	0
Febrile neutropenia	1 (2.8)	1 (2.8)	6 (12.5)	6 (12.5)

Abbreviation: TRAE, treatment‐related adverse event.

^a^ TRAEs that occurred in at least 20% of patients in either treatment group or TRAE of grade ≥ 3 that occurred in at least 5% of patients in either treatment group.

TRAEs of special interest for EV included skin reactions, peripheral neuropathy, and hyperglycemia. Treatment‐related rash occurred in 55.6% of patients who received EV and all events were of grade 3 or lower in severity (grade 1, *n* = 5; grade 2, *n* = 7; grade 3, *n* = 8) and in 12.5% of patients who received SC (grade 1, *n* = 2; grade 2, *n* = 3; grade 3, *n* = 1). Treatment‐related severe cutaneous adverse reaction occurred in 30.6% of EV‐treated patients (grade 1, *n* = 1; grade 2, *n* = 5; grade 3, *n* = 5) and 14.6% of SC‐treated patients (grade 1, *n* = 3; grade 2, *n* = 3; grade 3, *n* = 1); all events were grade 3 or lower in severity. Approximately half (52.8%, *n* = 19) of patients receiving EV experienced treatment‐emergent peripheral neuropathy, manifested primarily as peripheral sensory neuropathy (47.2%, *n* = 17). In patients receiving SC, 33.3% (*n* = 16) of patients experienced treatment‐emergent peripheral neuropathy, including 31.3% (*n* = 15) experiencing peripheral sensory neuropathy. All peripheral neuropathy events were mild or moderate (grade 1 or 2); no patient experienced grade ≥ 3 events in any treatment group. Peripheral neuropathy was considered treatment‐related in 19 (52.8%) patients receiving EV and 16 (33.3%) patients receiving SC. Treatment‐emergent hyperglycemia occurred in two patients each in the EV (5.6%; grade 2, *n* = 1 and grade 3, *n* = 1) and SC (4.2%; grade 3, *n* = 1 and grade 4, *n* = 1) groups; hyperglycemia was considered treatment‐related in both patients receiving EV and in none of the patients receiving SC. Rates of other TRAEs of special interest can be found in Table S1. Overall, the profiles, frequency, and severity of TRAEs were similar between the Japanese subpopulation and the overall EV‐301 population.

## 4 DISCUSSION

Given poor survival outcomes in patients with advanced UC who have progressed after platinum‐containing chemotherapy and PD‐1/L1 inhibitor treatment, there remains a large unmet need for efficacious and safe/tolerable treatments. In patients who have not progressed with first‐line platinum‐based chemotherapy, avelumab maintenance prolongs survival, including in Japanese patients with la/mUC.^23^, ^24^ The Japanese Urological Association guidelines for bladder cancer acknowledge results of KEYNOTE‐045 and recommend pembrolizumab in patients that have experienced recurrence or progression after first‐line platinum‐based chemotherapy and recommend avelumab as maintenance therapy following primary anticancer therapy. The lack of evidence‐based, effective, third‐line treatments remained a concern until Japan's Ministry of Health, Labour, and Welfare approved EV for unresectable UC that has progressed after cancer treatments.^12^, ^15^, ^16^, ^25^, ^26^ In a real‐world study of patients in Japan with metastatic UC undergoing second‐line chemotherapy (per physician decision and predominantly with a taxane‐based regimen [paclitaxel, ifosphamide, and nedaplatin] but also including platinum‐based regimens), median PFS was 4 months and OS was 9 months.^7^ It is known that the efficacy with PD‐1/L1 inhibitors is limited by their low (~20%) ORRs.^27^, ^28^ As avelumab is the only approved first‐line maintenance treatment and pembrolizumab remains the only approved agent for the second‐line treatment (after chemotherapy) of UC in Japan, additional effective and safe treatments remain a critical unmet need.^16^, ^19^, ^29^

Results of a previously published phase 1 study of EV in Japanese patients have shown many consistencies with the global population of the phase 1 trial. For example, in a prior phase 1 study of EV in Japanese patients, pharmacokinetic parameters appeared to be consistent with previously reported pharmacokinetic data in non‐Japanese patients, and antitumor activity was demonstrated based on an objective response rate of 35.3% and DCR of 76.5%.^30^, ^31^ In EV‐301, patients with la/mUC treated with EV had significant and clinically meaningful improvements in OS, PFS, and ORR compared with patients that received SC.^11^ Patients in Japan comprised approximately 15% (86 of 608 patients) of patients in the overall EV‐301 study population. Thus, findings in the majority population (i.e., patients in other countries or of White race) could have driven the efficacy results in the overall study population. In Japanese patients, who were representative of the general population of patients with UC,^4^ the risk of death was reduced by 56% in patients receiving EV compared with SC. Overall, efficacy results in the Japanese subgroup in EV‐301 were remarkably consistent with the OS and PFS, as well as confirmed ORR and DoR findings observed in the overall population.

No new safety signals were identified for patients in the Japanese subgroup in EV‐301, and safety findings were generally consistent with the overall population.^11^ Recent studies of various anticancer agents have shown differences in the frequency or profile of toxicities between Asian and Caucasian populations. In studies of metastatic prostate cancer or other advanced solid tumors in subgroups of Japanese patients, select AEs (i.e., hepatoxicity, hematologic toxicities, hyperglycemia) appeared higher in the Japanese subgroup versus the overall population.^20^, ^21^, ^32^ While the Japanese subgroup receiving EV seemed to appear to have a higher incidence of alopecia and dysgeusia, these findings were in line with safety findings from the phase 1 study.^30^ Possible reasons for the higher incidence of these findings remain unknown. Further investigation is needed. Japanese patients in EV‐301 had higher (≥10% difference of any grade TRAE occurring in at least 10% of patients in either treatment arm in the Japanese subgroup analysis) proportions of certain AEs, including dysgeusia (50.0% vs. 24.3%), peripheral sensory neuropathy (47.2% vs. 33.8%), decreased white blood cell count (25.0% vs. 5.4%), anemia (22.2% vs. 11.5%), and decreased neutrophil count (22.2% vs. 10.1%) compared with the overall EV‐301 population.^11^ However, EV tolerability seemed to be maintained in Japanese patients because differences in the rate of treatment discontinuation, dose reduction, and dose interruption between the overall EV‐301 population and Japanese patients appeared comparable. EV‐treated patients had a longer median duration of treatment compared with chemotherapy‐treated patients. Based on our subgroup analysis, we believe AEs observed with EV to be tolerable and manageable for Japanese patients with la/mUC. Should AEs occur with EV, treatment interruption, dose reduction, or treatment discontinuation could be considered in accordance with guidance.^22^ Our data demonstrate that patients receiving EV had no substantial differences in the frequency and safety profile between ethnic groups. However, the effect of randomization is not preserved in subgroups; therefore, caution should be warranted in interpreting these results. The number of patients included in this data set was relatively small and confirmation with a larger sample (i.e., real‐world studies) would further support these findings.

In conclusion, treatment after platinum chemotherapy and/or PD‐1/L1 inhibitor treatment with EV demonstrated longer OS compared with SC in previously treated patients with la/mUC in the Japanese subpopulation. Japanese patients receiving EV had longer time to disease progression or death, higher objective tumor response rate, and a lower rate of grade ≥ 3 TRAEs than those receiving SC. Based on the consistent and favorable benefit–risk profile across the EV‐301 populations, EV is a valuable addition to the treatment armamentarium for la/mUC in Japan with the potential to improve outcomes for patients with a life‐threatening malignancy.

## AUTHOR CONTRIBUTIONS

Thomas Powles, Jonathan E. Rosenberg, Daniel P. Petrylak, Maria Matsangou, Chunzhang Wu, Mary Campbell, and Mayumi Yamashiro had substantial contribution to study design. Nobuaki Matsubara, Junji Yonese, Takahiro Kojima, Haruhito Azuma, Hiroaki Matsumoto, Thomas Powles, Jonathan E. Rosenberg, and Daniel P. Petrylak contributed to the acquisition of study data. Maria Matsangou, CW, and MC contributed to the analysis of study data. Nobuaki Matsubara, Junji Yonese, Takahiro Kojima, Haruhito Azuma, Hiroaki Matsumoto, Thomas Powles, Jonathan E. Rosenberg, Daniel P. Petrylak, Maria Matsangou, Chunzhang Wu, Mary Campbell, and Mayumi Yamashiro contributed to the interpretation of study data.

## FUNDING INFORMATION

This study is sponsored by Astellas Pharma, Inc. and Seagen Inc.

## CONFLICT OF INTEREST

NM reports speakers' bureau for Janssen and Sanofi and research funding (institutional) from Janssen, AstraZeneca, Bayer, Roche, MSD, Taiho, Astellas, Amgen, Eisai, Eli Lilly, Takeda, Pfizer, and Chugai. TP reports grants and consulting fees from AstraZeneca, Roche, BMS, Exelixis, Ipsen, Merck, MSD < Novartis, Pfizer, Seattle Genetics, Merck Serono, Astellas, Johnson & Johnson, Eisai; support for attending meetings Roche, Pfizer, MSD, AstraZeneca, Ipsen. JER reports grants from Genentech/Roche, Bayer, AstraZeneca, and QED; consulting fees from Lilly, Merck, Roche/Genentech, Astellas, SeaGen, AstraZeneca, BMS, BioClin, QED, Pharmacyclics, GSK, Janssen, Boehringer Ingelheim, Pfizer, EMD Serono, Mirati, Immunomedics/Gilead, Tyra Biosciences, Infinity Pharmaceuticals, and Bayer; honoraria from EMD‐Serono, UpToDate, Research To Practice, Physicians Education Resource, and MJH Life Sciences. DPP reports grants for institutional support from Ada Cap (Advanced Accelerator Applications), Agensys Inc, Astellas, AstraZeneca, Bayer, BioXcel Therapeutics, Bristol Myers Squibb, Clovis Oncology, Eisai, Eli Lilly, Endocyte, Genentech, Gilead Sciences, Innocrin, MedImmune, Medivation, Merck, Mirati, Novartis, Pfizer, Progenics, Replimune, Roche, Sanofi Aventis, and Seattle Genetics; consulting fees from Ada Cap (Advanced Accelerator Applications), Amgen, Astellas, AstraZeneca, Bayer, Bicycle Therapeutics, Boehringer Ingelheim, Bristol Myers Squibb, Clovis Oncology, Eli Lilly, Exelixis, Gilead Sciences, Incyte, Ipsen, Janssen, Mirati, Monopteros, Pfizer, Pharmacyclics, Regeneron, Roche, Seattle Genetics, and Urogen; sold stock in Bellicum (sold 7/2020) and Tyme (sold 10/2019). MC is an employee of Seagen, Inc. MM, CW, and MY are employees of Astellas Pharma, Inc. No other authors have disclosures.

## ETHICAL APPROVAL STATEMENT

The EV‐301 trial received approval from independent institutional review boards and independent ethics committees and was conducted in accordance with the International Council for Harmonization guidelines for Good Clinical Practice and the Declaration of Helsinki. Written informed consent was obtained from patients prior to trial entry.

## Supporting information


Table S1

## Data Availability

Researchers may request access to anonymized participant level data, trial level data and protocols from Astellas sponsored clinical trials at www.clinicalstudydatarequest.com. For the Astellas criteria on data sharing see: https://clinicalstudydatarequest.com/Study‐Sponsors/Study‐Sponsors‐Astellas.aspx.
