# Supplementary material for: Japanese subgroup analysis of EV‐301: An open‐label, randomized phase 3 study to evaluate enfortumab vedotin versus chemotherapy in subjects with previously treated locally advanced or metastatic urothelial carcinoma
Source: Cancer Med. 2022 Sep 2;12(3):2761–71. doi: 10.1002/cam4.5165 (PMC9939146; doi:10.1002/cam4.5165)
Supplement: Supplementary file 1 — Table S1 [file CAM4-12-2761-s001.pdf]

Supporting Information

List of Investigators

Country	Primary Investigator
**JAPAN**	Abe, Takashige; Ebara, Shin; Fukasawa, Satoshi; Hashimoto, Yasuhiro; Inokuchi, Junichi; Kanayama, Hiroomi; Kawasaki, Yoshihide; Kitamura, Hiroshi; Kobayashi, Takashi; Kondo, Yukihiro; Masumori, Naoya; Mizuno, Ryuichi; Nishimura, Kazuo; Obara, Wataru; Sugimoto, Mikio; Takamoto, Atsushi; Tomita, Yoshihiko; Uemura, Hiroji; Yokomizo, Akira; Yoshimura, Kazuhiro

Investigators are listed in alphabetical order by country and last name.

Supplemental Table

Table S1. Treatment-Related Adverse Events of Special Interest† in the Japanese Subgroup (Safety Population)

Event, n (%)	Enfortumab Vedotin (N=36)	Standard Chemotherapy (N=48)
**Skin reactions‡**	**21 (58.3)**	**12 (25.0)**
Rash	20 (55.6)	6 (12.5)
Severe cutaneous adverse reaction§	11 (30.6)	7 (14.6)
**Peripheral neuropathy**	**19 (52.8)**	**16 (33.3)**
Sensory events¶	18 (50.0)	16 (33.3)
Motor events	1 (2.8)	0
**Ocular disorders**	**2 (5.6)**	**2 (4.2)**
Dry eye	2 (5.6)	1 (2.1)
Blurred vision	0	1 (2.1)
Corneal disorders	0	0
**Infusion-related reactions**	**7 (19.4)**	**3 (6.3)**
Systemic events	6 (16.7)	3 (6.3)
Local events	1 (2.8)	0
Infusion site reactions	1 (2.8)	0
Extravasation site reactions	1 (2.8)	0
**Hyperglycemia**	**2 (5.6)**	**0**

†Events represent listings by Preferred Term and may include Sponsor Specific Query/Customized Medical Queries (SSQ/CMQ) or Standard MedDRA Queries.
‡Indicates rash or severe cutaneous adverse reactions.
§Composite Standard MedDRA Query High Level Term of severe cutaneous adverse reactions including: stomatitis, drug eruption, conjunctivitis, dermatitis bullous, skin exfoliation, blister, erythema multiforme, exfoliative rash, fixed eruption, mouth ulceration, pemphigus, and toxic skin eruption.
¶Represents any peripheral neuropathy sensory events (SSQ/CMQ) including: peripheral sensory neuropathy, neuropathy peripheral, paresthesia, polyneuropathy, hypoesthesia, neurotoxicity, dysesthesia, gait disturbance, burning sensation, neuralgia, and sensory loss.
